# Sp1 phosphorylation by ATM downregulates BER and promotes cell elimination in response to persistent DNA damage

**DOI:** 10.1093/nar/gkx1291

**Published:** 2017-12-27

**Authors:** Sally C Fletcher, Claudia P Grou, Arnaud J Legrand, Xin Chen, Kalle Soderstrom, Mattia Poletto, Grigory L Dianov

**Affiliations:** 1 Department of Oncology, CRUK & MRC Oxford Institute for Radiation Oncology, University of Oxford, Old Road Campus Research Building, Oxford OX3 7DQ, UK; 2 Department of Marine Technology, College of Ocean, Nantong University, Nantong, Jiangsu, 226007, China; 3 Nuffield Department of Orthopaedics, Rheumatology and Musculoskeletal Sciences, Botnar Research Centre, University of Oxford, Oxford OX3 7LD, UK; 4 Institute of Cytology and Genetics, Russian Academy of Sciences, Lavrentyeva 10 Novosibirsk 630090, Russian Federation; 5 Novosibirsk State University, Pirogova 2, Novosibirsk 630090, Russian Federation

## Abstract

ATM (ataxia-telangiectasia mutated) is a central molecule for DNA quality control. Its activation by DNA damage promotes cell-cycle delay, which facilitates DNA repair prior to replication. On the other hand, persistent DNA damage has been implicated in ATM-dependent cell death via apoptosis; however, the mechanisms underlying this process remain elusive. Here we find that, in response to persistent DNA strand breaks, ATM phosphorylates transcription factor Sp1 and initiates its degradation. We show that Sp1 controls expression of the key base excision repair gene *XRCC1*, essential for DNA strand break repair. Therefore, degradation of Sp1 leads to a vicious cycle that involves suppression of DNA repair and further aggravation of the load of DNA damage. This activates transcription of pro-apoptotic genes and renders cells susceptible to elimination via both apoptosis and natural killer cells. These findings constitute a previously unrecognized ‘gatekeeper’ function of ATM as a detector of cells with persistent DNA damage.

## INTRODUCTION

Failure to preserve genome stability underlies the decline of every organism through pathophysiological processes such as ageing, neurodegeneration and cancer. For this reason, the cellular genome is constantly guarded against DNA lesions generated by both exogenous and endogenous mutagens. In order to maintain genome stability, cells exploit a number of DNA repair systems. Amongst these, the DNA base excision repair (BER) pathway constitutes the frontline defense against endogenously-generated DNA damage including DNA base lesions and single strand breaks (SSBs) (1,2). BER is robust pathway that, under normal circumstances, is sufficient to cope with endogenous DNA damage. However, low BER efficiency can occur as a result of malfunction of BER components or overload of cellular repair capacity by acute DNA damage. This can either lead to cell death, or result in accumulation of mutations and induction of tumorigenesis (3–5). It is still poorly understood how cells decide to trigger apoptosis and eliminate potentially dangerous cells, rather than attempting DNA repair of excessively damaged DNA. The ATM (ataxia-telangiectasia mutated) protein is the primary candidate for this role of decision maker (6). In fact, ATM’s kinase activity has been shown to respond to a wide range of genome-threatening lesions including DNA single and double strand breaks (7,8) and to mobilize a cascade of phosphorylation events that delay cell cycle, providing additional time for DNA repair prior to replication (9). At the same time, ATM has been implicated in the initiation of programmed cell death in response to DNA damage (10,11). Despite this, although ATM-dependent cell cycle delay is known to be mainly accomplished through effectors such as p53 and p21 (12), the mechanisms driving an ATM-dependent switch to apoptosis are unclear.

Among a number of proteins that are phosphorylated by ATM in response to DNA damage, transcription factor Sp1 has been shown to regulate the expression of multiple genes (13–15), including cellular components involved in apoptosis (16–18). While ATM-dependent phosphorylation of Sp1 at serine 101 has been previously documented (13–15), the biological role of this modification remains unclear. Here we report that, in response to persistent DNA strand breaks, ATM phosphorylates transcription factor Sp1 at serine 101, initiating its proteasomal-dependent degradation. We also find that Sp1 controls the expression of the key BER gene *XRCC1* and that degradation of Sp1 decreases DNA repair capacity and aggravates the load of DNA damage. This feeds a vicious cycle that further supports Sp1 degradation. Furthermore, we demonstrate that downregulation of Sp1 upon DNA damage primes fibroblasts to apoptosis and to elimination by natural killer (NK) cells. We suggest that this mechanism allows the detection of potentially pre-cancerous cells bearing persistent DNA strand breaks, prompting their removal either through apoptosis or via the innate immune system.

## MATERIALS AND METHODS

### Cell culture and drug treatments

Normal human TIG-1 fibroblasts were from the Coriell Institute Cell Repository (AG06173). Cells were cultured in Dulbecco’s modified Eagle’s medium low glucose (Life Technologies) supplemented with 15% foetal bovine serum (FBS) at 37°C in a humidified atmosphere with 5% CO_2_. The Nishi NK cell line has been previously described, and is derived from the peripheral blood mononuclear cells of a boy with chronic active Epstein–Barr virus infection complicated with NK leukaemia. The phenotype of this NK leukaemia is: CD94 / NKG2A and LIR-1 / ILT-2 positive, but CD3, α βTCR, γ δTCR, KIR3DL1, KIR2DL1, KIR2DL2, KIR2DS1, KIR2DS2 negative. CD16 expression is low (19). NK cells were grown in IMDM GlutaMAX™ medium (Life Technologies) supplemented with 10% FBS, 2% heat-inactivated human serum (Sigma-Aldrich), 100 IU/ml penicillin, 100 μg/ml streptomycin and 10 ng/ml recombinant human IL-15 (PeproTech) at 37°C in a humidified atmosphere with 7.5% CO_2_. Cells were routinely checked for mycoplasma. H_2_O_2_, camptothecin, cycloheximide, the Chk1 inhibitor (UCN-01) and midostaurin were from Sigma. Zeocin was from Life Technologies. Mithramycin A and MG132 were from Enzo Life Sciences. The ATM inhibitors (KU55933 and KU60019), the DNA-PK inhibitor (Inhibitor III) and staurosporine were obtained from Millipore, while the Chk2 inhibitor (CCT 241533) was from Tocris. The ataxia telangiectasia and Rad3-related (ATR) inhibitor (VE-821) was a kind gift from Dr Anderson Ryan (University of Oxford). Navitoclax (ABT-263) was purchased from Cayman Chemical.

### Cell viability assays

Cell viability was assessed using resazurin (Sigma). For co-culture experiments, TIG-1 cells were treated as described and a suspension of NK cells was aliquoted onto adherent fibroblasts at the indicated NK:TIG-1 ratio. Cell cytotoxicity was assessed after co-incubation by washing off NK cells and evaluating the viability of fibroblasts using a resazurin assay.

### Comet assays, immunostaining and high-throughput microscopy

Alkaline comet assays were carried out as previously described (7). Immunostaining and high-throughput microscopy were carried out as described in (20).

### Protein expression and purification

The plasmid pN3-Sp1FL, containing full-length Sp1 was a gift from Guntram Suske (Addgene plasmid #24543). A pET-28a plasmid expressing His_(6)_-tagged recombinant Sp1 was generated by sub-cloning the Sp1 cDNA from pN3-Sp1FL. Protein expression was carried out in Rosetta™ *Escherichia coli* cells and recombinant Sp1 was purified under denaturing conditions (6 M guanidine hydrochloride) using a HisTrap column (GE Healthcare). Recombinant Sp1 was refolded over 96 h by sequential dialysis against 10 mM Tris–HCl, pH 7.5, 200 mM NaCl, 50 μM ZnSO_4_, 0.4 M L-Arginine, 5% glycerol for 48 h, followed by 10 mM Tris–HCl, pH 7.5, 200 mM NaCl, 5% glycerol for further 48 h.

### *In vitro* phosphorylation assays

Phosphorylation reactions were carried out by combining recombinant Sp1 (500 ng) and active recombinant ATM (100 ng - Millipore) in phosphorylation buffer (50 mM HEPES pH 7.5, 50 mM KCl, 10 mM MgCl_2_, 10 mM MnCl_2_, 1 mM adenosine triphosphate (ATP), 1 mM dithiothreitol (DTT) and 5% glycerol). Reactions were incubated for 2 h at 30°C and halted by adding sodium dodecyl sulphate-polyacrylamide gel electrophoresis loading buffer.

### *In vitro* ligation assays

Nuclear cell extracts were prepared as described previously (21). Ligation assays were carried out using 1 μg of nuclear extract essentially as described in (22), with minor modifications. Briefly, reactions were performed in 50 mM Tris–HCl pH 7.5, 10 mM MgCl_2_, 10 mM DTT, 1 mM ATP at 23°C for the indicated time; the oligonucleotide substrate (50 nM) has been described (22) and was 5′-labeled with IRDye^®^800 (IDT). Reactions were halted with 96% formamide and 10 mM EDTA and analysed by electrophoresis on a 20% denaturing polyacrylamide gel. The percentage of substrate converted to product was determined by using an Odyssey image analysis system (Li-Cor Biosciences).

### Luciferase assays and chromatin immuno-precipitation (ChIP)

Luciferase assays and chromatin immuno-precipitation (ChIP) assays were carried out essentially as in (20). Sp1 binding to the *XRCC1* promoter (−196 to −17 bp) was assessed by ChIP using the primers reported in the Supplementary Methods. For luciferase assay, a reporter plasmid containing the *XRCC1* promoter was generated by amplifying the genomic region upstream the *XRCC1* gene (−3969 to +174 bp) and by cloning it into a pGL3 plasmid (Promega) using standard molecular biology techniques.

### Electrophoretic mobility shift assays (EMSA)

Electrophoretic mobility shift assays (EMSAs) were carried out essentially as in (20). An *XRCC1* promoter probe (−145 to −128 bp) was obtained by annealing a 5′-IRDye^®^800-labeled oligonucleotide (5′-GTGTGGCGGAGGGAGGCGGGGCTGGAGGAAACG-3′ (Integrated DNA Technologies)), with its complementary sequence. An Sp1 consensus probe (5′-ATTCGATCGGGGCGGGGCGAGC-3′) was used as cold competitor in order to confirm binding specificity.

### Flow cytometry

For flow cytometry analyses, cells were harvested by trypsinization and apoptosis was measured using the AnnexinV-FITC Apoptosis Detection kit (Abcam) as per manufacturer’s instructions. Samples were acquired using a Becton-Dickinson FACSCalibur™ instrument and data analysis was carried out using either BD CellQuest Pro or FlowJo.

### Statistical analyses

Statistical analyses were performed with the two-tailed Student’s *t*-test using either Microsoft Excel or SPSS (IBM). Sample size is indicated for each experiment.

## RESULTS

### Direct phosphorylation of Sp1 at serine 101 by ATM in response to DNA damage

**Figure 1.**
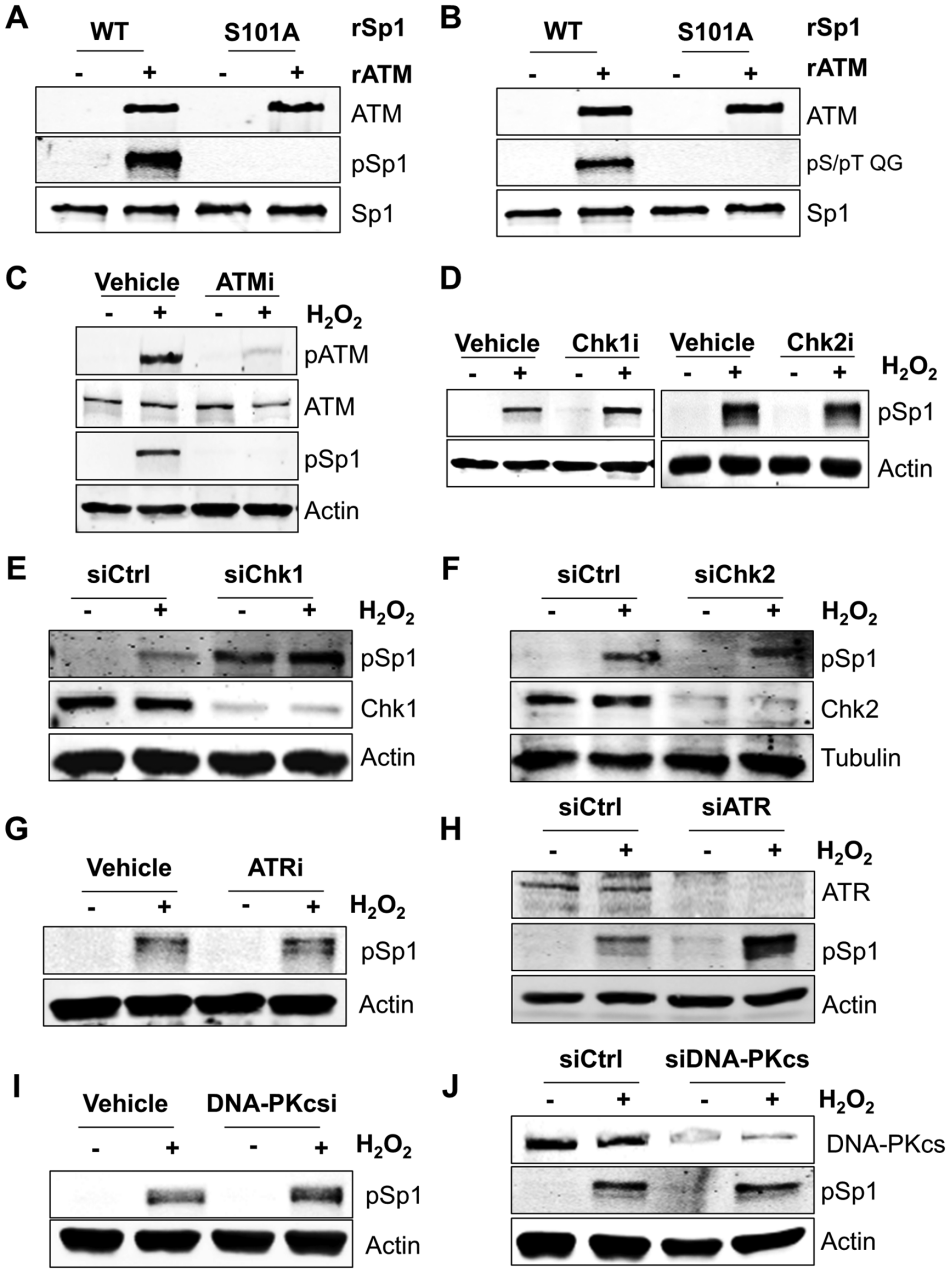
ATM is the main kinase responsible for serine 101 phosphorylation of Sp1. (**A** and **B**) Representative *in vitro* kinase assay using recombinant ATM (rATM) and wild-type (WT) or Ser101-Ala mutant (S101A) recombinant Sp1 proteins (rSp1). ATM can phosphorylate WT Sp1 but is unable to phosphorylate the S101A mutant. Reactions were probed with either the phosphorylated Ser101-specific antibody (A) or an ATM/ATR substrate-specific pS/pT QG antibody (B). (**C**) Representative Western blot analysis on TIG-1 cells showing Sp1 phosphorylation at Ser101 upon H_2_O_2_ treatment (200 μM, 1 h). Loss of phosphorylation occurs in the presence of ATM inhibitor (ATMi: KU55933, 10 μM, 2 h prior to H_2_O_2_). (**D**) Representative Western blot analysis as in (C), in the presence of Chk1 inhibitor (UCN-01, 10 μM for 2 h prior to H_2_O_2_) or Chk2 inhibitor (CCT 241533, 3 nM for 2 h prior to H_2_O_2_). (**E** and **F**) Representative Western blot analysis as in (C), on cells depleted of either Chk1 (E) or Chk2 (F) using siRNA. Phosphorylation of Sp1 is still detected after treatment with H_2_O_2_. (**G**) Same as in (C), but cells were treated with an ATR inhibitor (VE-821, 1 μM for 2 h prior to H_2_O_2_) (**H**) Same as in (E), but cells were treated with an ATR siRNA. (**I**) Same as in (C), but cells were treated with a DNA-PKcs inhibitor (DNA-PK Inhibitor III, 10 μM for 2 h prior to H_2_O_2_). (**J**) Same as in (E), but cells were treated with DNA-PKcs siRNA. Data information: In (A–J) either actin or tubulin was used as loading control.

Although a number of studies have demonstrated that Sp1 phosphorylation at Ser101 depends on ATM (13–15), it is not clear whether Sp1 phosphorylation is accomplished by ATM itself or can also be mediated by other ATM-activated kinases. In order to unequivocally demonstrate that ATM is responsible for direct phosphorylation of Sp1 at Ser101 we employed an *in vitro* kinase assay using purified proteins. Co-incubation of active recombinant ATM and recombinant purified Sp1 showed that Sp1 is robustly phosphorylated by ATM at Ser101 (Figure 1A). Importantly, an Sp1 mutant in which Ser101 was changed into alanine (S101A) could not be phosphorylated *in vitro* (Figure 1A), confirming the specificity of the antibody used in this study. Furthermore, when the phosphorylation reaction was probed with an antibody specific to the phospho-ATM/ATR substrate motif (pS/pT QG), the Sp1 S101A mutant did not show any phosphorylation (Figure 1B), suggesting that Ser101 is the major site phosphorylated by ATM under these conditions. Consistent with previous findings (14,15), Sp1 phosphorylation was readily induced by H_2_O_2_ treatment in normal human fibroblasts (Figure 1C). Modification of Sp1 was prevented by inhibition of ATM kinase activity (Figure 1C), confirming that Ser101 could be modified by either ATM or by another kinase downstream to ATM *in vivo*. Using specific kinase inhibitors we demonstrated that Sp1 phosphorylation at Ser101 was not dependent on either Chk1 or Chk2 (Figure 1D) activity. These observations were also substantiated by siRNA-mediated knockdown of either Chk1 (Figure 1E) or Chk2 (Figure 1F), leading us to conclude that these kinases are not involved in Sp1 phosphorylation at Ser101 in response to DNA damage. Additionally, we checked if this phosphorylation was dependent on other members of the phosphatidylinositol 3-kinase family involved in the DNA damage response (DDR), namely ATR and DNA-dependent protein kinase catalytic subunit (DNA-PKcs). Neither ATR inhibition (Figure 1G), nor siRNA-mediated knockdown (Figure 1H) abrogated Ser101 phosphorylation upon treatment with H_2_O_2_. Similarly, neither inhibition (Figure 1I), nor siRNA-mediated knockdown of DNA-PKcs (Figure 1J) led to a loss of Ser101 phosphorylation, indicating that Ser101 phosphorylation was not dependent on either ATR or DNA-PKcs. These data demonstrate that, in response to DNA damage, ATM is the major kinase responsible for direct Sp1 phosphorylation at Ser101.

### Unrepaired DNA strand breaks promote phosphorylation-dependent degradation of Sp1

**Figure 2.**
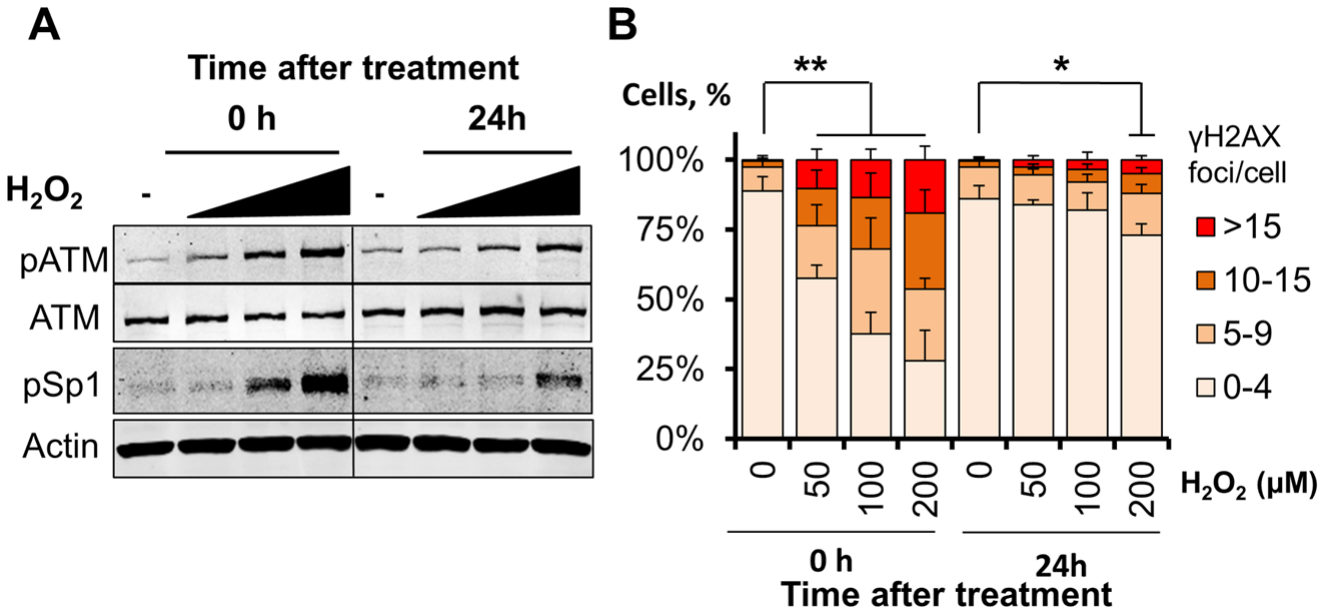
Phosphorylation of Sp1 at Ser101 correlates with the amount of DNA damage. (**A**) Representative Western blot analysis on TIG-1 cells showing dose-dependent induction of ATM phosphorylation at Ser1981 and Sp1 phosphorylation upon treatment with H_2_O_2_ (50–100–200 μM, 1 h) and release for either 0 or 24 h. Actin was used as loading control. (**B**) Quantification of γH2AX foci in TIG-1 cells treated as in (A). Foci were scored using high-throughput microscopy. At least 500 cells/condition were analyzed. The histogram reports the foci distribution as mean ± SD from three independent experiments and confirms persistency of DNA double strand breaks at the highest H_2_O_2_ dose 24 h after treatment *P < 0.05; **P < 0.01.

**Figure 3.**
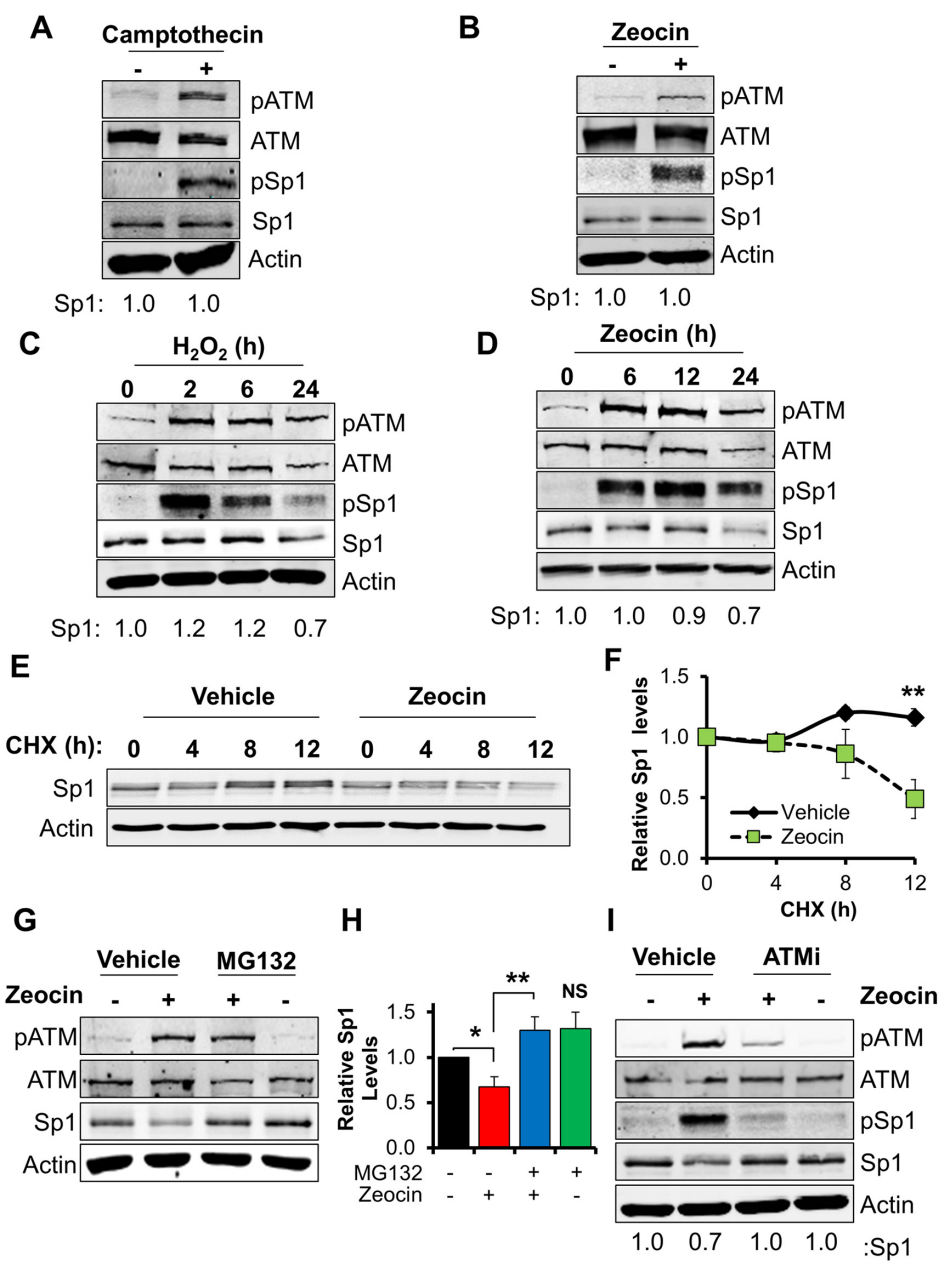
Unrepaired DNA strand breaks promote phosphorylation-dependent degradation of Sp1. (**A** and **B**) Representative Western blot analysis on TIG-1 cells treated with camptothecin (10 μM, 1 h) (A), or zeocin (50 μg/ml, 2 h) (B) and showing ATM and Sp1 phosphorylation. (**C** and **D**) Representative time-course on TIG-1 cells treated with H_2_O_2_ (200 μM) (C), or zeocin (50 μg/ml) (D) showing time-dependent ATM and Sp1 phosphorylation, followed by degradation of Sp1. (**E**) Representative cycloheximide time-course on TIG-1 cells (50 μg/ml) showing reduced Sp1 half-life upon zeocin treatment (50 μg/ml). (**F**) Densitometric quantification of the data presented in panel (E). Data are expressed as mean ± SD from three independent experiments and confirm that Sp1 downregulation upon zeocin is due to increased protein turnover. (**G**) Representative Western blot analysis on TIG-1 cells showing that Sp1 downregulation upon zeocin treatment (50 μg/ml, 24 h) can be prevented by inhibition of proteasomal activity with MG132 (10 μM, 6 h). (**H**) Densitometric quantification of the data presented in panel (F). Data are expressed as mean ± SD from three independent experiments and confirm that Sp1 downregulation upon zeocin is proteasome dependent. (**I**) Representative Western blot analysis on TIG-1 cells showing decreased amount of Sp1 upon zeocin treatment (50 μg/ml, 24 h) and recovery upon co-incubation with an ATM inhibitor (ATMi: KU60019, 10 μM, 24 h). NS: not significant; *P < 0.05; **P < 0.01. Data information: In (A–I) actin was used as loading control.

Human fibroblasts challenged with H_2_O_2_ are known to activate ATM in a dose-dependent manner (14) and this is accompanied by accumulation of Ser101-phosphorylated Sp1 (Figure 2A). We observe here a striking correlation between ATM activation, the Sp1 phosphorylation pattern and the amount of DNA double strand breaks arising after a short H_2_O_2_ treatment, as measured by scoring γH2AX foci (Figure 2B). Notably, dose-dependent Sp1 phosphorylation observed immediately after H_2_O_2_ treatment (Figure 2A, left-hand side) correlated with the amount of DNA double strand breaks generated (Figure 2B, left-hand side). Phosphorylation persisted 24 h after treatment (Figure 2A, right-hand side) as a consequence of unrepaired DNA strand breaks produced at the highest H_2_O_2_ dose tested (Figure 2B, right-hand side). Consistent with this, other DNA strand-break inducing agents such as topoisomerase I inhibitor camptothecin and radiomimetic zeocin were also able to trigger robust phosphorylation of Sp1 after a brief treatment (Figure 3A and B).

The functional consequences of Sp1 phosphorylation are poorly understood. To address this gap in our knowledge we investigated the fate of phosphorylated Sp1 after persistent DNA damage induced by prolonged exposure of cells to H_2_O_2_ or zeocin. Interestingly, we observed a time-dependent reduction of both phosphorylated and total Sp1 (Figure 3C and D), suggesting that Sp1 phosphorylation at serine 101 may trigger destabilization of the protein. Furthermore, cycloheximide treatment demonstrated reduced Sp1 half-life after treatment with zeocin (Figure 3E and F). In addition, inhibition of proteasome activity by MG132 prevented zeocin-induced Sp1 depletion suggesting persistent DNA damage may negatively affect Sp1 stability (Figure 3G and H). In order to demonstrate that ATM kinase activity is ultimately responsible for the modulation of Sp1 protein levels, we incubated cells with zeocin in the presence of an ATM inhibitor. Inhibition of ATM kinase activity prevented Sp1 downregulation induced by zeocin (Figure 3I), suggesting that DNA damage leads to ATM-dependent phosphorylation of Sp1, followed by proteasomal degradation of the transcription factor.

### Depletion of Sp1 impairs repair of endogenous DNA damage via BER

Having established that in response to unrepaired DNA strand breaks Sp1 undergoes ATM-dependent phosphorylation and degradation, we next sought to understand the consequences of a reduction in Sp1 levels on the ability of cells to repair DNA.

**Figure 4.**
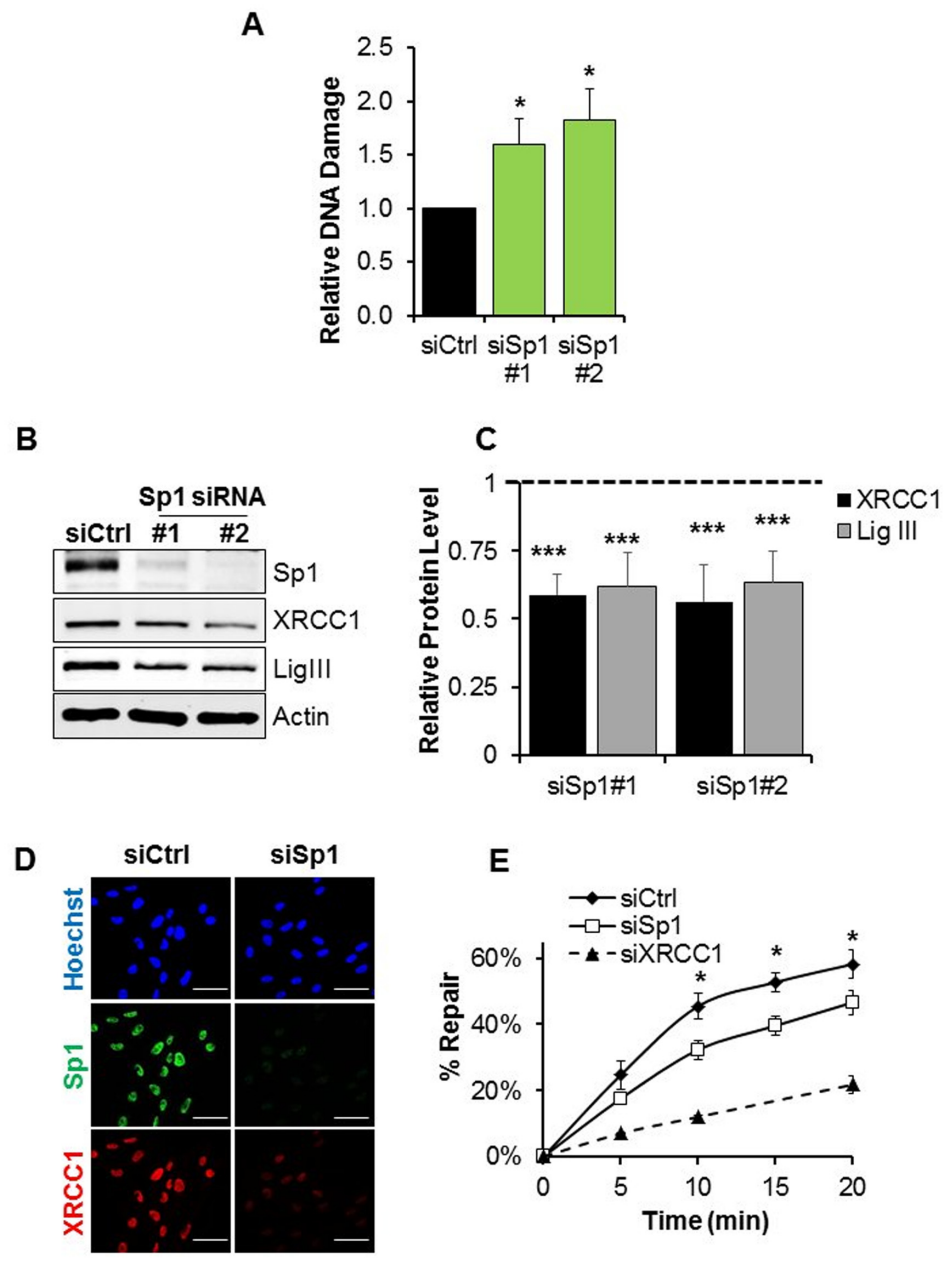
Depletion of Sp1 leads to XRCC1 downregulation and impairs repair of endogenous DNA damage via BER. (**A**) Alkaline comet assay on TIG-1 cells 72 h after Sp1 depletion showing accumulation of DNA damage (*N* = 3). Relative DNA damage represents the percentage of DNA in the comet tail normalized to the level in siCtrl cells. (**B**) Representative western blot analysis on cells treated as in (A). Sp1 depletion results in a reduction in XRCC1 and LigIII protein levels. Actin was used as loading control. (**C**) Densitometric quantification of data presented in panel (B) confirming Sp1 depletion results in a reduction in XRCC1 and LigIII protein levels (*N=* 4). (**D**) Representative micrographs on cells treated as in (A) showing downregulation of XRCC1 upon Sp1 knockdown. Scale bars 50 μm. (**E**) *In vitro* assay measuring the nick ligation activity in Sp1- and XRCC1-depleted nuclear cell extracts. Sp1 downregulation impairs cellular nick ligation efficiency (*N* = 3). Data information: In (A, C and E), data are reported as mean ± SD from the indicated number (*N*) of independent experiments *P < 0.05; ***P < 0.001.

Alkaline comet assays showed that siRNA-mediated depletion of Sp1 leads to an increased amount of endogenously generated DNA strand breaks, even in the absence of external stimuli (Figure 4A). As BER is the frontline mechanism against endogenous DNA lesions, this phenotype pointed to a potential deficiency in this DNA repair pathway. In order to test this, following Sp1 depletion we measured the level of XRCC1 and DNA ligase III, key proteins involved in the repair of endogenous SSBs. Consistent with the observation of spontaneous accumulation of DNA strand breaks (Figure 4A), we found that both proteins were downregulated after Sp1 knockdown (Figure 4B–D; Supplementary Figure S1). This resulted in a mild, but significant deficiency in SSB repair in Sp1-depleted cells, as confirmed by *in vitro* ligation assays using nuclear cell extracts and a nick-containing oligonucleotide duplex (Figure 4E).

### Basal transcription of *XRCC1* is modulated by Sp1

**Figure 5.**
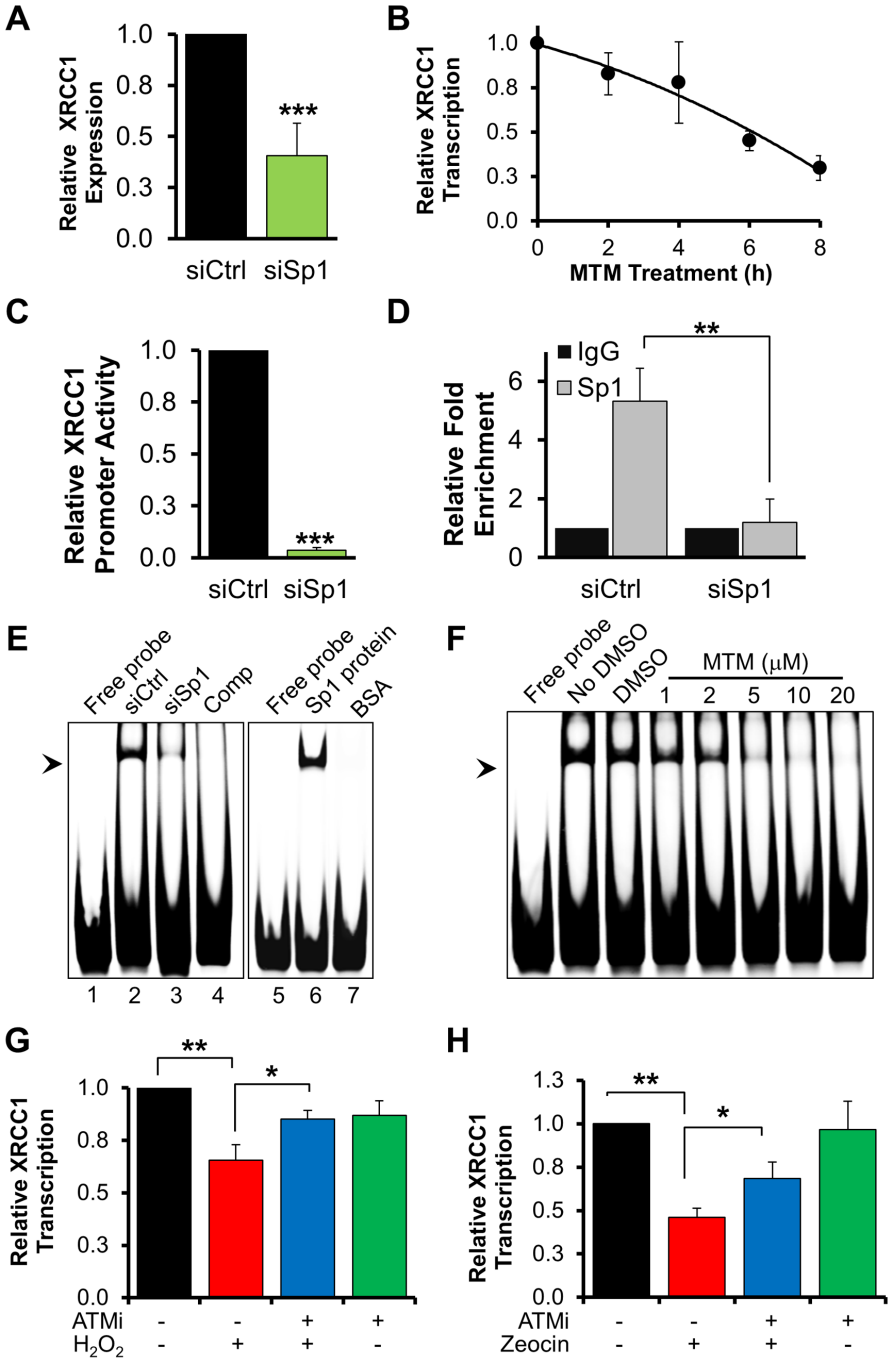
Basal transcription of *XRCC1* is modulated by Sp1. (**A**) qPCR analysis showing reduction in *XRCC1* transcription following Sp1 depletion (*N* = 4). (**B**) qPCR analysis of XRCC1 transcript levels in TIG-1 cells treated with the Sp1 inhibitor mithramycin A (MTM, 1 μM) for the indicated time. The level of XRCC1 transcript decreases in a time-dependent manner (*N* = 3). (**C**) Histogram showing reduction in *XRCC1* promoter activity in cells depleted of Sp1, as measured by luciferase assays (*N* = 4). (**D**) ChIP analysis on TIG-1 cells assessing ability of Sp1 to bind to the *XRCC1* proximal promoter. Sp1 is enriched at the *XRCC1* promoter under basal conditions. Enrichment is lost upon Sp1 depletion. Results are expressed as fold enrichment relative to the unspecific IgG (*N* = 3). (**E**) Representative EMSA measuring Sp1 binding activity to an *XRCC1* proximal promoter probe. *Left:* TIG-1 cells were treated with the indicated siRNA and nuclear extracts were used to assess Sp1 binding activity. The arrowhead indicates formation of a Sp1-containing protein–DNA complex, which is lost upon Sp1 depletion. Comp: unlabeled Sp1 competitor sequence shows specificity of protein–DNA complex formation. *Right*: EMSA using recombinant Sp1 protein and assessing its binding activity to the *XRCC1* promoter probe. BSA was used as negative control. (**F**) Representative EMSA measuring Sp1 binding activity to an *XRCC1* proximal promoter probe in the presence of Sp1 inhibitor (MTM). Increasing concentrations of Sp1 inhibitor prevent formation of the protein–DNA complex. (**G** and **H**) qPCR analysis of XRCC1 transcript levels in TIG-1 cells treated with H_2_O_2_ (125 μM, 72 h) (G) or zeocin (50 μg/ml, 72 h) (H) in presence of an ATM inhibitor (ATMi: KU60019, 10 μM 72 h), as indicated. Suppression of *XRCC1* transcription upon DNA damage is ATM dependent (*N* = 3). Data information: In (A, B, C, D, G and H) data are reported as mean ± SD from the indicated number (*N*) of independent experiments *P < 0.05; **P < 0.01; ***P< 0.001.

As depletion of Sp1 decreased XRCC1 protein levels (Figure 4B and C), we next tested whether Sp1 might be directly involved in the regulation of *XRCC1* gene expression. Several lines of evidence were in support of this. First, XRCC1 mRNA levels were reduced in both Sp1-depleted cells (Figure 5A) and in cells treated with the Sp1 inhibitor mithramycin A (Figure 5B). Secondly, using luciferase assays we measured the transcriptional activity of the genomic region spanning ∼4 kb upstream from the *XRCC1* transcription start site. These experiments confirmed that Sp1 modulates cellular activity of the *XRCC1* promoter, as depletion of Sp1 resulted in a significant reduction of the reporter signal (Figure 5C). Additionally, ChIP assays allowed us to confirm that Sp1 binds a region between −196 and −17 bp within the *XRCC1* promoter (Figure 5D). Finally, using an oligonucleotide containing the putative Sp1 binding site in the *XRCC1* promoter in electrophoretic mobility shift assays (EMSAs) we detected Sp1 binding activity in nuclear cell extracts (Figure 5E). EMSA experiments allowed us to narrow the Sp1 binding site to a region between −145 and −128 bp within the *XRCC1* promoter. Importantly, under these experimental conditions Sp1 was likely the only factor able to bind the promoter probe, as siRNA-mediated depletion of Sp1 resulted in a loss of binding activity in cell extracts (Figure 5E, lane 3). Binding was also affected by supplementing the nuclear extracts with the Sp1 inhibitor mithramycin A (Figure 5F). Moreover, Sp1 binding to the *XRCC1* probe was completely abolished in the presence of an Sp1 consensus competitor oligonucleotide (Figure 5E, lane 4), while recombinant purified Sp1 was sufficient to achieve efficient binding of the probe (Figure 5E, lane 6). Our findings strongly suggest that Sp1 modulates basal transcription of *XRCC1* in normal human fibroblasts.

As we demonstrated that Sp1 degradation occurred in an ATM-dependent manner in response to persistent unrepaired DNA strand breaks (Figure 3I), we checked whether this could affect *XRCC1* expression. In line with our hypothesis, XRCC1 mRNA levels were significantly decreased in cells treated with H_2_O_2_ or zeocin, and this could be prevented by inhibition of ATM (Figure 5G and H). This indicates that ATM-dependent Sp1 phosphorylation and degradation modulate *XRCC1* expression in response to persistent DNA strand breaks.

We conclude that Sp1 is responsible for modulation of *XRCC1* gene expression and that activation of the ATM/Sp1 axis in response to DNA damage has a negative impact on *XRCC1* transcription and BER efficiency.

### The ATM/Sp1 cross-talk primes cells bearing persistent DNA damage to apoptosis and accelerates their elimination through NK cells

Our data demonstrate that persistent DNA lesions activate ATM, which phosphorylates Sp1 and triggers a cellular response that leads to suppression of DNA repair and further DNA damage. We hypothesized that this somewhat counterintuitive process could be a mechanism of amplification of a signal that allows cells with persistent DNA strand breaks to be flagged for irreversible elimination. Although previous studies indicated that ATM promotes programmed cell death (23,24) and that BER deficiency can lead to apoptosis (4,25) the mechanism involved was not clear.

**Figure 6.**
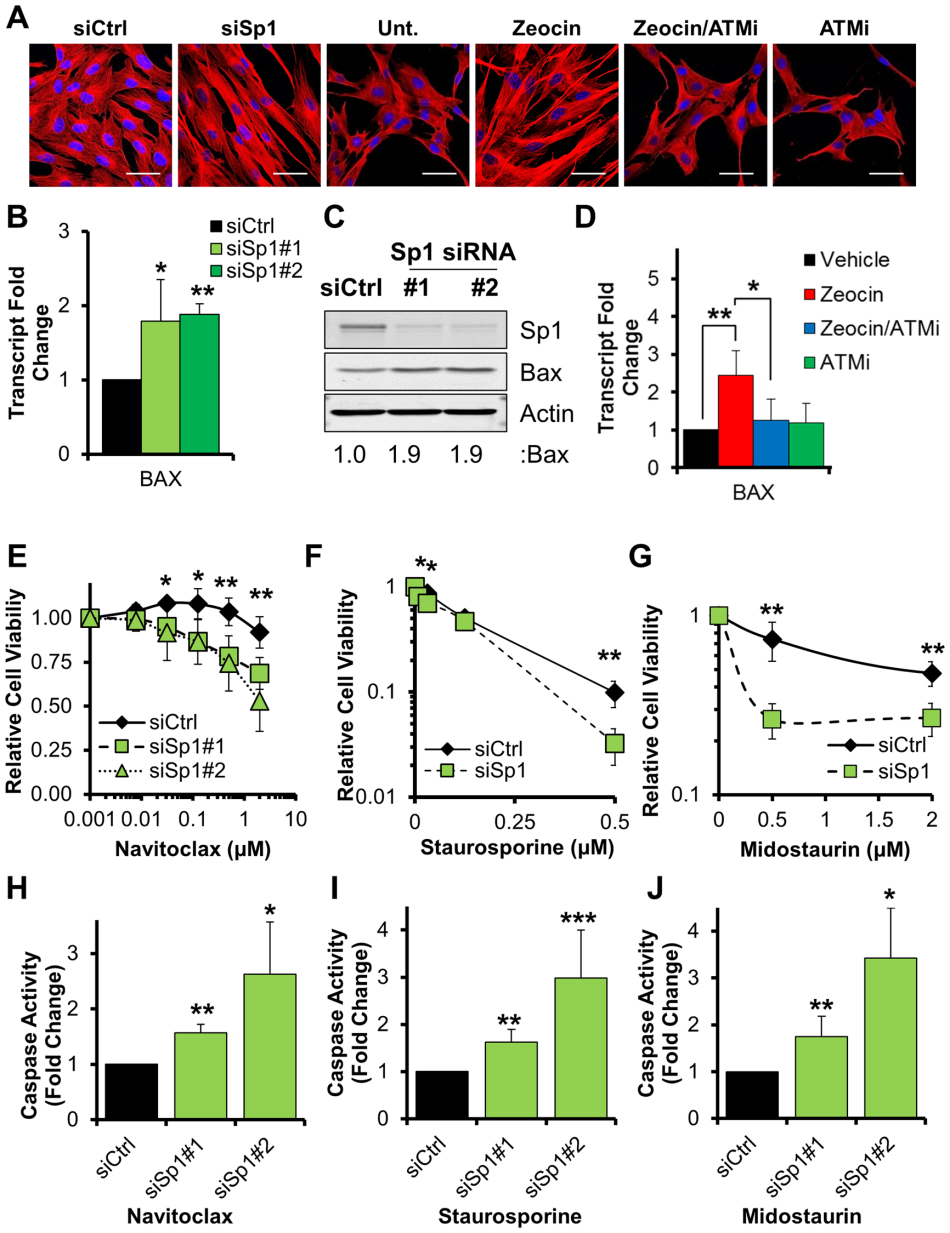
Downregulation of Sp1 primes fibroblasts to apoptosis. (**A**) Representative micrographs from TIG-1 cells treated with either control siRNA (siCtrl), Sp1-targeting siRNA (siSp1) or zeocin (50 μg/ml, 72 h). An ATM inhibitor (ATMi: KU60019, 10 μM 72 h) was added where indicated. Both Sp1 depletion and zeocin treatments result in cytoskeleton contraction, as assessed by α-tubulin staining (red). The phenotype is reversed by ATM inhibition. Hoechst (blue) was used to stain nuclei. Scale bars: 50 μm. (**B**) qPCR analysis assessing transcription of *Bax* in TIG-1 cells treated with the indicated siRNA (*N* = 3). (**C**) Representative Western blot analysis on TIG-1 cells showing increased BAX protein levels upon Sp1 depletion. (**D**) qPCR analysis assessing transcription of *Bax* in fibroblasts treated with zeocin (50 μg/ml, 6 h). An ATM inhibitor (ATMi: KU60019, 10 μM) was added where indicated. Induction of *Bax* is completely prevented by ATM inhibition (*N* = 4). (**E**–**G**) Sensitivity of TIG-1 cells depleted of Sp1 to navitoclax (E), staurosporine (F) or midostaurin (G). Cells were treated with increasing doses of the indicated drug 48 h after siRNA transfection. Cell viability was measured using resazurin 48 h (E and G) or 24 h (F) after treatment (*N* = 3). (**H**–**J**) Caspase 3/7 activity assays showing increased caspase activity in cells treated with navitoclax (2 μM, 48 h) (H), staurosporine (125 nM, 24 h) (I) or midostaurin (8 μM, 48 h) upon Sp1-depletion (J). Bars report the fold change in caspase activity, normalized to drug-treated siCtrl cells (*N* = 4). Data information: In (B–I) data are reported as mean ± SD from the indicated number (*N*) of independent experiments *P < 0.05; **P < 0.01; ***P < 0.001. In (C) actin was used as loading control and densitometric quantification is reported below the blot.

**Figure 7.**
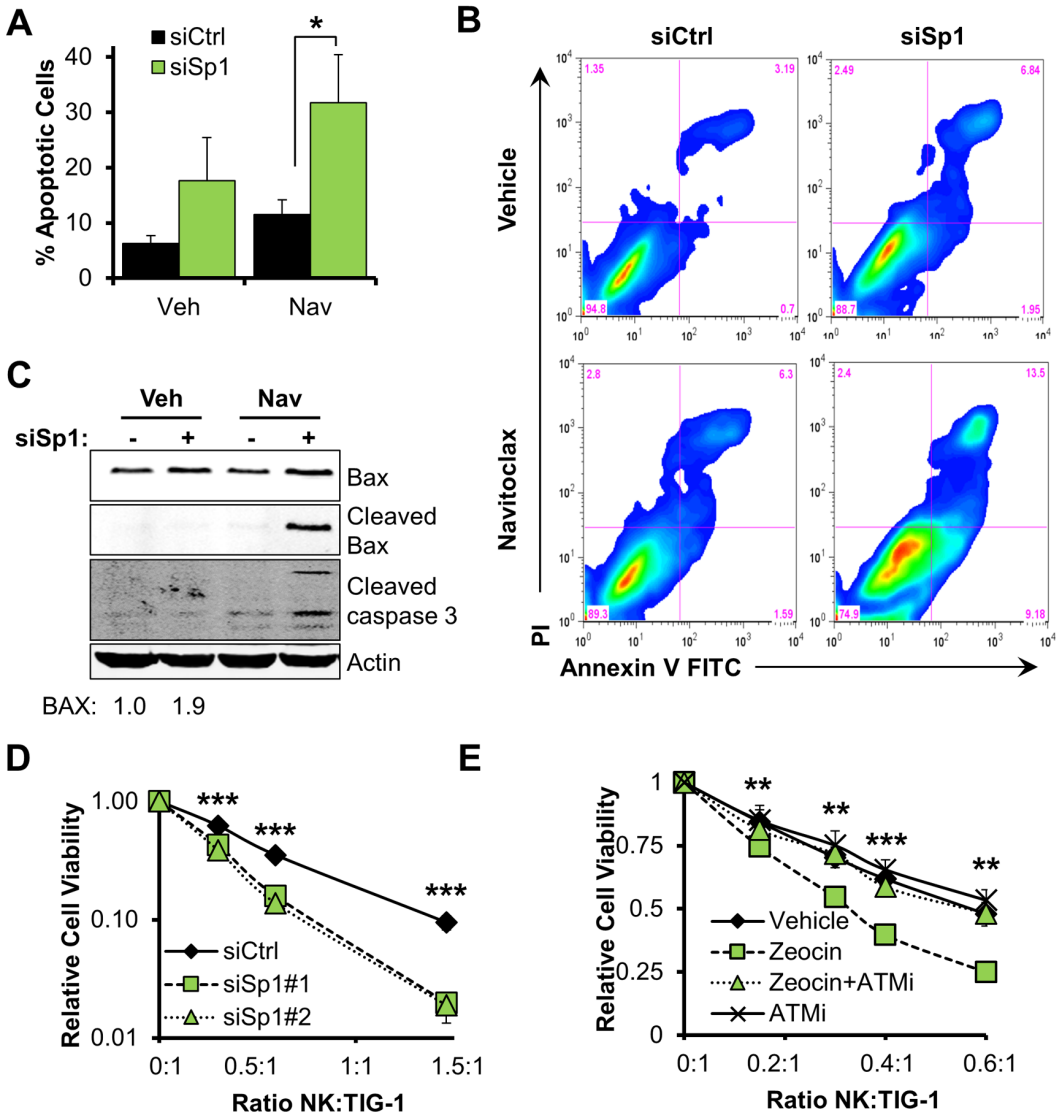
Downregulation of Sp1 primes fibroblasts to apoptosis and elimination by NK cells. (**A** and **B**) Apoptosis in cells depleted of Sp1 upon treatment with navitoclax. Cells were transfected with the indicated siRNA and treated with either DMSO (Veh) or navitoclax (Nav: 2 μM, 48 h) before Annexin V/PI staining and FACS analysis (*N* = 4). A representative density plot showing increased early- and late-apoptotic cells (*bottom right* and *top right* quadrant, respectively) is shown (B). (**C**) Representative Western blot analysis on cells treated as in (A) and showing cleaved caspase 3 and BAX in cells depleted of Sp1 upon treatment with navitoclax (Nav). Quantification of total BAX is reported at the bottom of the gel; actin was used as loading control. (**D**) Survival of TIG-1 fibroblasts upon incubation with NK cells. TIG-1 cells were treated with the indicated siRNA for 48 h before co-culture with NK cells at the indicated ratio for a further 24 h. Cell viability was measured using resazurin. Data show dose-response curves representative of three independent biological replicates. (**E**) Survival of TIG-1 fibroblasts upon incubation with NK cells. Cells were treated as indicated for 48 h, followed by co-incubation with NK cells for further 14 h. Cell viability was measured using resazurin. Data show dose-response curves representative of three independent biological replicates. Data information: In (A, D and E) data are reported as mean ± SD from the indicated number (*N*) of independent experiments *P < 0.05; **P < 0.01; ***P < 0.001.

In order to test our hypothesis we performed a series of experiments. First, we examined cell morphology after either Sp1 depletion, or zeocin treatment. We found that both conditions led to noticeable changes in cell morphology inlcuding an intensified cytoskeleton network, elongated fusiform cellular shape and shrinkage of cellular extremities, as assessed by staining for the cytoskeleton protein α-tubulin (Figure 6A). Importantly, this phenotype was dependent on ATM activity, as ATM inhibition completely restored cellular morphology upon zeocin treatment (Figure 6A). Altogether, these changes are consistent with intense cellular stress, and are reminiscent of the very early morphological profile observed in apoptotic fibroblasts (26). Following this conclusion, we investigated whether cells expressing low levels of Sp1 could be in a pre-apoptotic state. This idea was supported by qPCR analyses that confirmed transcriptional upregulation of canonical pro-apoptotic genes including *BAX* and PUMA (*BBC3*) in Sp1-depleted fibroblasts (Figure 6B and Supplementary Figure S2A). Consistent with qPCR data, Sp1-depleted cells expressed increased protein levels of BAX (Figure 6C). Importantly, ATM inhibition also effectively prevented zeocin-induced upregulation of pro-apoptotic genes (Figure 6D and Supplementary Figure S2B), consistent with a leading role for ATM in the signaling process. Moreover, Sp1-depleted cells showed greater sensitivity to apoptosis induced by the BH3-mimetic navitoclax (Figure 6E), or pro-apoptotic agents such as staurosporine (Figure 6F) and midostaurin (Figure 6G). In fact, cell treatment with these agents induced full-blown apoptosis specifically in Sp1-depleted cells, as demonstrated by caspase3/7 activation assays (Figure 6H–J), annexin V/propidium iodide staining (Figure 7A and B) and cleavage of caspase 3 (Figure 7C). Furthermore, apoptosis regulator BAX underwent cleavage specifically in Sp1-knockdown cells upon treatment with navitoclax (Figure 7C). As BAX cleavage has been proposed to boost apoptosis (27,28), we concluded that cells expressing low levels of Sp1 are primed to apoptosis and that pro-apoptotic agents promptly trigger programmed cell death in these fibroblasts.

Our data suggest that, at the cellular level, downregulation of Sp1 in response to persistent DNA strand breaks primes cells to elimination. In a fully developed organism, however, the innate immune response is tasked with the elimination of genetically unstable host cells. In particular, NK cells have been shown to respond to oncogenic stress and DNA damage, whereby upregulation of activating ligands in target cells has been proposed to be dependent on ATM/ATR signaling (29,30). We reasoned that fibroblasts bearing unrepaired DNA strand breaks and expressing lower amounts of Sp1 could be susceptible to recognition and elimination via NK cells. Consistent with our hypothesis, co-culture experiments showed that Sp1-depleted fibroblasts were indeed more susceptible to cell death induced by NK cells (Figure 7D). Fibroblast hypersensitivity to NK-mediated elimination was also observed when DNA damage was induced using zeocin (Figure 7E) and crucially, this phenotype was completely dependent on ATM’s kinase activity (Figure 7E).

In conclusion, we have discovered a mechanism whereby activation of ATM by persistent DNA strand breaks leads to downregulation of transcription factor Sp1. This promotes BER downregulation, further accumulation of DNA damage and primes fibroblasts to apoptosis. These conditions promote elimination of cells exposed to persistent DNA damage by the innate immune system.

## DISCUSSION

The inherent chemical instability of DNA, as well as intra- and extra-cellular mutagens are the main sources of genomic DNA lesions, including DNA strand breaks (1). BER is the major DNA repair pathway dealing with endogenous DNA lesions and, under normal circumstances, BER capacity curbs accumulation of SSBs, limiting spontaneous formation of DSBs. However, while accumulation of DNA damage can occur in response to acute genotoxic stress, unrepaired DNA strand breaks can also be present under pathophysiological circumstances where impaired BER is not able to promptly repair endogenous DNA damage, leading to genetic instability (7,31–34). For this reason, control mechanisms must be in place to promote elimination of cells harboring unrepaired DNA strand breaks to ultimately prevent genomic instability and finally malignant transformation.

The main question addressed by this study is what are the molecular mechanisms that detect cells deficient in the repair of endogenous DNA lesions and direct them towards elimination?

**Figure 8.**
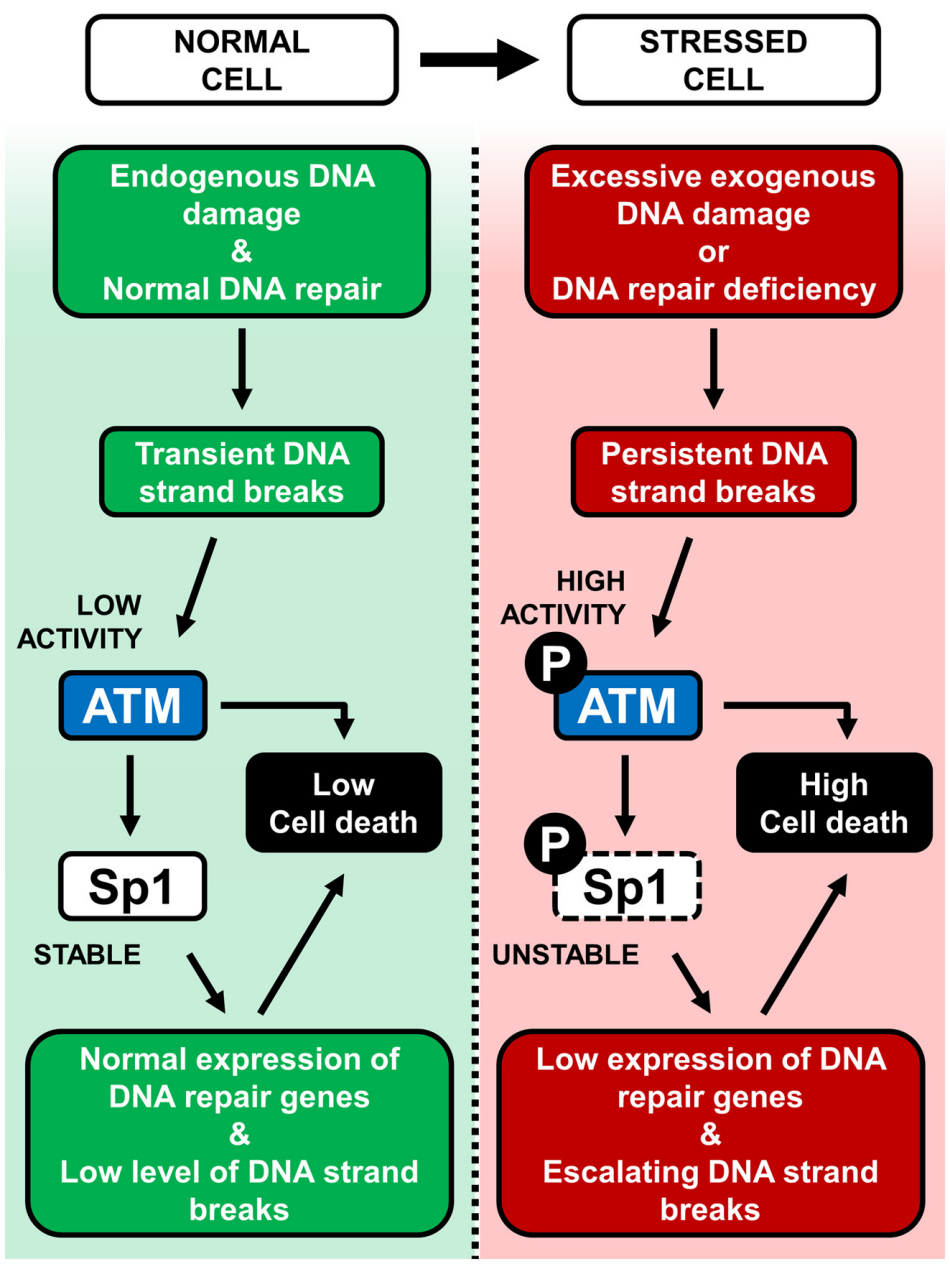
Graphical summary of the model supported by this study. Left: in a normal cell, endogenous DNA damage is kept under control by DNA repair mechanisms. Low ATM activity ensures stable Sp1 protein levels, promoting efficient expression of DNA repair genes and low levels of cell death. Right: genomically stressed cells can arise under conditions of excessive exogenous DNA damage, or DNA repair deficiency. This leads to persistent DNA strand breaks and ATM activation, which triggers Sp1 phosphorylation and degradation. In this situation the cellular load of DNA strand breaks is aggravated by decreased expression of DNA repair genes, leading to further ATM phosphorylation, activation of a pro-apoptotic cascade and death through apoptosis or cell elimination.

We propose a model whereby downregulation of transcription factor Sp1 in response to a signal radiating from ATM contributes to the elimination of potentially pre-cancerous cells harboring persistent DNA damage. We note that phosphorylation of ATM, and the phosphorylated form of transcription factor Sp1, are barely detectable under unstressed conditions (Figures 1, 2 and 3). At this stage, basal transcription of the *XRCC1* gene, and consequently BER capacity, are clearly sufficient to maintain genomic stability (Figure 8, left). However, in response to accumulation of DNA strand breaks, ATM phosphorylates and destabilizes transcription factor Sp1. In turn, this leads to downregulation of *XRCC1* transcription, decreased levels of DNA ligase III and reduction in BER capacity. This mechanism leads to accumulation of further DNA strand breaks, resulting in a self-accelerating cycle (Figure 8, right). This kind of scenario may represent a response to excessive acute DNA damage, or to BER failure. In both cases this will result in cell elimination either through apoptosis or by components of the innate immune system. We believe this represents a protective pro-survival mechanism to ensure the removal of cells with persistent unrepaired DNA strand breaks.

Sp1 Ser101 phosphorylation in response to DNA damage has been previously described in a number of studies (13–15) although the specific trigger for Ser101 phosphorylation was not known. We show that this occurs directly via ATM (Figure 1) in response to genome-threatening lesions including both SSBs and DSBs (7,8). These findings led us to hypothesise that DNA strand breaks in general are likely the major trigger for Ser101 phosphorylation. However, the inherent cross-talk between DDR kinases, and complex nature of DNA damage induced by agents such as zeocin means that we cannot exclude the involvement of other triggers, including stalled replication forks, or a role for other kinases in specific cellular contexts. Although the occurrence of Ser101 phosphorylation is well-documented (13–15), its precise function has remained elusive. This study addresses this gap in our knowledge, linking Ser101 phosphorylation with Sp1 protein stability. Our data suggest that Sp1 destabilization occurs via the proteasome (Figure 3F). However, the exact mechanism is unclear, such as whether Sp1 degradation is linked to ubiquitylation; we were unable to detect ubiquitylated Sp1 protein after DNA damage (data not shown). E3 ligase RNF4 and βTrCP, a component of SCF ubiquitin ligase complexes have previously been linked to Sp1 degradation (35,36). However, our attempts to prevent Sp1 downregulation after persistent DNA damage by targeting either RNF4 or βTrCP with siRNAs were unsuccessful (data not shown). Moreover, type, dose and duration of DNA damage are likely important considerations for determining the kinetics of the Sp1 response. Given that Sp1 destabilization in TIG-1 normal human fibroblasts did not occur immediately after genotoxic insult, but only when DNA damage persisted (Figure 3A–D), this suggests dependency on the cellular load of unrepaired DNA strand breaks. Decreased Sp1 protein levels after H_2_O_2_ treatment has previously been observed in HeLa cells (37). However, this occurred using a shorter but higher dose of H_2_O_2,_ thus supporting the hypothesis that the cellular DNA damage load is crucial for determining Sp1 stability. Moreover, the differential response time between fibroblasts and HeLa cells suggests that cell type is also an important determinant of Sp1 stability after DNA damage. Therefore, further study to fully elucidate the mechanism behind Sp1 degradation is warranted.

We demonstrated that loss of Sp1 is associated with increased sensitivity to cell elimination (Figure 7). Immune surveillance in whole organisms by components of the innate immune system including NK cells, constitutes an important defense against tumorigenesis by eliminating stressed, damaged or tumorigenic cells. Recognition of such cells is proposed to occur through upregulation of cytotoxic NK cell receptor NKG2D (NK group 2, member D) ligands which include the MIC and ULPBP protein families (29). Interestingly, expression of some activating NKG2D ligands is proposed to depend on the ATM/ATR signaling axis (29,30,38). It is therefore tempting to speculate that NK cells recognize and eliminate fibroblasts harboring persistent unrepaired DNA strand breaks through ATM-dependent upregulation of NKG2D ligands.

The consequences of DNA damage are inexorably linked to the severity of the lesion. It remains unknown, however, how cells transition between pro-survival and pro-death responses as a consequence of unrepairable DNA lesions. Several lines of evidence indicate that the ATM kinase performs a key role in this process (6); multiple studies highlight the involvement of ATM in apoptosis in response to DNA damage (10,11,39) and emerging evidence has linked ATM with promoting immune surveillance (29). Our study supports a role for ATM in the pro-survival/pro-death decision-making process through ATM-dependent destabilization of Sp1. Of note, ATM has been suggested to undergo selective pressure for inactivation in cancer (40), while loss of ATM function correlates with the occurrence of malignancies (41–43); this could explain how genetically unstable cells might escape this control mechanism, leading to cancer emergence.

While we demonstrate that this protective mechanism exists in normal untransformed fibroblasts, it is of interest to understand whether it remains active in cancer cells, particularly where ATM function is not impaired. It raises the question whether it is possible to target this mechanism using chemotherapeutic drugs to induce elimination of cancerous cells. Moreover, given the sensitivity to apoptosis observed in Sp1-depleted cells, it is tempting to speculate whether low-Sp1 expressing tumors might show sensitivity to pro-apoptotic agents. Clearly, the potential exploitation of this system requires further study.

Moreover, the existence of a similar mechanism involving downregulation of DNA repair to enable selective cell killing has recently been proposed (44). In their study, Ponath and Kaina suggested that monocytes expressing lower levels of BER components could be eliminated by their differentiated counterpart, macrophages, when these cells generate a reactive oxygen species burst during the inflammatory response (44).

In summary, we believe that the molecular mechanism we propose could explain how physiological populations of healthy cells are maintained in an organism, by detecting and eliminating cells with DNA repair defects.

## Supplementary Material

Supplementary Data

## References

[1] Lindahl T. Instability and decay of the primary structure of DNA. Nature. 1993; 362:709–715. 8469282 10.1038/362709a0

[2] Dianov G.L., Hubscher U. Mammalian base excision repair: the forgotten archangel. Nucleic Acids Res. 2013; 41:3483–3490. 23408852 10.1093/nar/gkt076 PMC3616742

[3] Horton J.K., Watson M., Stefanick D.F., Shaughnessy D.T., Taylor J.A., Wilson S.H. XRCC1 and DNA polymerase beta in cellular protection against cytotoxic DNA single-strand breaks. Cell Res. 2008; 18:48–63. 18166976 10.1038/cr.2008.7 PMC2366203

[4] Ochs K., Sobol R.W., Wilson S.H., Kaina B. Cells deficient in DNA polymerase beta are hypersensitive to alkylating agent-induced apoptosis and chromosomal breakage. Cancer Res. 1999; 59:1544–1551. 10197627

[5] Unnikrishnan A., Raffoul J.J., Patel H.V., Prychitko T.M., Anyangwe N., Meira L.B., Friedberg E.C., Cabelof D.C., Heydari A.R. Oxidative stress alters base excision repair pathway and increases apoptotic response in apurinic/apyrimidinic endonuclease 1/redox factor-1 haploinsufficient mice. Free Radic. Biol. Med. 2009; 46:1488–1499. 19268524 10.1016/j.freeradbiomed.2009.02.021 PMC2677124

[6] Roos W.P., Thomas A.D., Kaina B. DNA damage and the balance between survival and death in cancer biology. Nat. Rev. Cancer. 2016; 16:20–33. 26678314 10.1038/nrc.2015.2

[7] Khoronenkova S.V., Dianov G.L. ATM prevents DSB formation by coordinating SSB repair and cell cycle progression. Proc. Natl. Acad. Sci. U.S.A. 2015; 112:3997–4002. 25775545 10.1073/pnas.1416031112 PMC4386361

[8] Bakkenist C.J., Kastan M.B. DNA damage activates ATM through intermolecular autophosphorylation and dimer dissociation. Nature. 2003; 421:499–506. 12556884 10.1038/nature01368

[9] Shiloh Y. ATM: expanding roles as a chief guardian of genome stability. Exp. Cell Res. 2014; 329:154–161. 25218947 10.1016/j.yexcr.2014.09.002

[10] Karlseder J., Broccoli D., Dai Y., Hardy S., de Lange T. p53- and ATM-dependent apoptosis induced by telomeres lacking TRF2. Science. 1999; 283:1321–1325. 10037601 10.1126/science.283.5406.1321

[11] Paulson Q.X., Pusapati R.V., Hong S., Weaks R.L., Conti C.J., Johnson D.G. Transgenic expression of E2F3a causes DNA damage leading to ATM-dependent apoptosis. Oncogene. 2008; 27:4954–4961. 18469863 10.1038/onc.2008.138

[12] Shiloh Y., Ziv Y. The ATM protein kinase: regulating the cellular response to genotoxic stress, and more. Nat. Rev. Mol. Cell Biol. 2013; 14:197–210. 23847781

[13] Iwahori S., Yasui Y., Kudoh A., Sato Y., Nakayama S., Murata T., Isomura H., Tsurumi T. Identification of phosphorylation sites on transcription factor Sp1 in response to DNA damage and its accumulation at damaged sites. Cell. Signal. 2008; 20:1795–1803. 18619531 10.1016/j.cellsig.2008.06.007

[14] Olofsson B.A., Kelly C.M., Kim J., Hornsby S.M., Azizkhan-Clifford J. Phosphorylation of Sp1 in response to DNA damage by ataxia telangiectasia-mutated kinase. Mol. Cancer Res. 2007; 5:1319–1330. 18171990 10.1158/1541-7786.MCR-07-0374

[15] Hau P.M., Deng W., Jia L., Yang J., Tsurumi T., Chiang A.K., Huen M.S., Tsao S.W. Role of ATM in the formation of the replication compartment during lytic replication of Epstein-Barr virus in nasopharyngeal epithelial cells. J. Virol. 2015; 89:652–668. 25355892 10.1128/JVI.01437-14 PMC4301132

[16] Grande L., Bretones G., Rosa-Garrido M., Garrido-Martin E.M., Hernandez T., Fraile S., Botella L., de Alava E., Vidal A., Garcia del Muro X. Transcription factors Sp1 and p73 control the expression of the proapoptotic protein NOXA in the response of testicular embryonal carcinoma cells to cisplatin. J. Biol. Chem. 2012; 287:26495–26505. 22718761 10.1074/jbc.M112.376319 PMC3410991

[17] Li H., Zhang Y., Strose A., Tedesco D., Gurova K., Selivanova G. Integrated high-throughput analysis identifies Sp1 as a crucial determinant of p53-mediated apoptosis. Cell Death Differ. 2014; 21:1493–1502. 24971482 10.1038/cdd.2014.69 PMC4131181

[18] Hirose T., Horvitz H.R. An Sp1 transcription factor coordinates caspase-dependent and -independent apoptotic pathways. Nature. 2013; 500:354–358. 23851392 10.1038/nature12329 PMC3748152

[19] Cerboni C., Mousavi-Jazi M., Wakiguchi H., Carbone E., Karre K., Soderstrom K. Synergistic effect of IFN-gamma and human cytomegalovirus protein UL40 in the HLA-E-dependent protection from NK cell-mediated cytotoxicity. Eur. J. Immunol. 2001; 31:2926–2935. 11592068 10.1002/1521-4141(2001010)31:10<2926::aid-immu2926>3.0.co;2-2

[20] Poletto M., Legrand A.J., Fletcher S.C., Dianov G.L. p53 coordinates base excision repair to prevent genomic instability. Nucleic Acids Res. 2016; 44:3165–3175. 26773055 10.1093/nar/gkw015 PMC4838360

[21] Markkanen E., Fischer R., Ledentcova M., Kessler B.M., Dianov G.L. Cells deficient in base-excision repair reveal cancer hallmarks originating from adjustments to genetic instability. Nucleic Acids Res. 2015; 43:3667–3679. 25800737 10.1093/nar/gkv222 PMC4402536

[22] McNeill D.R., Narayana A., Wong H.K., Wilson D.M. 3rd Inhibition of Ape1 nuclease activity by lead, iron, and cadmium. Environ. Health Perspect. 2004; 112:799–804. 15159209 10.1289/ehp.7038 PMC1241995

[23] Westphal C.H., Rowan S., Schmaltz C., Elson A., Fisher D.E., Leder P. atm and p53 cooperate in apoptosis and suppression of tumorigenesis, but not in resistance to acute radiation toxicity. Nat. Genet. 1997; 16:397–401. 9241281 10.1038/ng0897-397

[24] Duchaud E., Ridet A., Stoppa-Lyonnet D., Janin N., Moustacchi E., Rosselli F. Deregulated apoptosis in ataxia telangiectasia: association with clinical stigmata and radiosensitivity. Cancer Res. 1996; 56:1400–1404. 8640831

[25] Roos W.P., Kaina B. DNA damage-induced cell death: from specific DNA lesions to the DNA damage response and apoptosis. Cancer Lett. 2013; 332:237–248. 22261329 10.1016/j.canlet.2012.01.007

[26] Moodley Y.P., Caterina P., Scaffidi A.K., Misso N.L., Papadimitriou J.M., McAnulty R.J., Laurent G.J., Thompson P.J., Knight D.A. Comparison of the morphological and biochemical changes in normal human lung fibroblasts and fibroblasts derived from lungs of patients with idiopathic pulmonary fibrosis during FasL-induced apoptosis. J. Pathol. 2004; 202:486–495. 15095276 10.1002/path.1531

[27] Cao X., Deng X., May W.S. Cleavage of Bax to p18 Bax accelerates stress-induced apoptosis, and a cathepsin-like protease may rapidly degrade p18 Bax. Blood. 2003; 102:2605–2614. 12816867 10.1182/blood-2003-01-0211

[28] Wood D.E., Newcomb E.W. Cleavage of Bax enhances its cell death function. Exp. Cell Res. 2000; 256:375–382. 10772810 10.1006/excr.2000.4859

[29] Cerboni C., Fionda C., Soriani A., Zingoni A., Doria M., Cippitelli M., Santoni A. The DNA damage response: a common pathway in the regulation of NKG2D and DNAM-1 ligand expression in normal, infected, and cancer cells. Front. Immunol. 2014; 4:508. 24432022 10.3389/fimmu.2013.00508 PMC3882864

[30] Raulet D.H., Guerra N. Oncogenic stress sensed by the immune system: role of natural killer cell receptors. Nat. Rev. Immunol. 2009; 9:568–580. 19629084 10.1038/nri2604 PMC3017432

[31] Bauer M., Goldstein M., Christmann M., Becker H., Heylmann D., Kaina B. Human monocytes are severely impaired in base and DNA double-strand break repair that renders them vulnerable to oxidative stress. Proc. Natl. Acad. Sci. U.S.A. 2011; 108:21105–21110. 22160723 10.1073/pnas.1111919109 PMC3248544

[32] Narciso L., Fortini P., Pajalunga D., Franchitto A., Liu P., Degan P., Frechet M., Demple B., Crescenzi M., Dogliotti E. Terminally differentiated muscle cells are defective in base excision DNA repair and hypersensitive to oxygen injury. Proc. Natl. Acad. Sci. U.S.A. 2007; 104:17010–17015. 17940040 10.1073/pnas.0701743104 PMC2040456

[33] Weissman L., Jo D.G., Sorensen M.M., de Souza-Pinto N.C., Markesbery W.R., Mattson M.P., Bohr V.A. Defective DNA base excision repair in brain from individuals with Alzheimer's disease and amnestic mild cognitive impairment. Nucleic Acids Res. 2007; 35:5545–5555. 17704129 10.1093/nar/gkm605 PMC2018628

[34] Higo T., Naito A.T., Sumida T., Shibamoto M., Okada K., Nomura S., Nakagawa A., Yamaguchi T., Sakai T., Hashimoto A. DNA single-strand break-induced DNA damage response causes heart failure. Nat. Commun. 2017; 8:15104. 28436431 10.1038/ncomms15104 PMC5413978

[35] Wei S., Chuang H.C., Tsai W.C., Yang H.C., Ho S.R., Paterson A.J., Kulp S.K., Chen C.S. Thiazolidinediones mimic glucose starvation in facilitating Sp1 degradation through the up-regulation of beta-transducin repeat-containing protein. Mol. Pharmacol. 2009; 76:47–57. 19372209 10.1124/mol.109.055376 PMC2701453

[36] Wang Y.T., Yang W.B., Chang W.C., Hung J.J. Interplay of posttranslational modifications in Sp1 mediates Sp1 stability during cell cycle progression. J. Mol. Biol. 2011; 414:1–14. 21983342 10.1016/j.jmb.2011.09.027

[37] Chuang J.Y., Chang W.C., Hung J.J. Hydrogen peroxide induces Sp1 methylation and thereby suppresses cyclin B1 via recruitment of Suv39H1 and HDAC1 in cancer cells. Free Radic. Biol. Med. 2011; 51:2309–2318. 22036763 10.1016/j.freeradbiomed.2011.10.001

[38] Gasser S., Orsulic S., Brown E.J., Raulet D.H. The DNA damage pathway regulates innate immune system ligands of the NKG2D receptor. Nature. 2005; 436:1186–1190. 15995699 10.1038/nature03884 PMC1352168

[39] Lee Y., Chong M.J., McKinnon P.J. Ataxia telangiectasia mutated-dependent apoptosis after genotoxic stress in the developing nervous system is determined by cellular differentiation status. J. Neurosci. 2001; 21:6687–6693. 11517258 10.1523/JNEUROSCI.21-17-06687.2001 PMC6763074

[40] Negrini S., Gorgoulis V.G., Halazonetis T.D. Genomic instability—an evolving hallmark of cancer. Nat. Rev. Mol. Cell Biol. 2010; 11:220–228. 20177397 10.1038/nrm2858

[41] Paull T.T. Mechanisms of ATM Activation. Annu. Rev. Biochem. 2015; 84:711–738. 25580527 10.1146/annurev-biochem-060614-034335

[42] Russell R., Perkhofer L., Liebau S., Lin Q., Lechel A., Feld F.M., Hessmann E., Gaedcke J., Guthle M., Zenke M. Loss of ATM accelerates pancreatic cancer formation and epithelial-mesenchymal transition. Nat. Commun. 2015; 6:7677. 26220524 10.1038/ncomms8677 PMC4532798

[43] Feng X., Li H., Dean M., Wilson H.E., Kornaga E., Enwere E.K., Tang P., Paterson A., Lees-Miller S.P., Magliocco A.M. Low ATM protein expression in malignant tumor as well as cancer-associated stroma are independent prognostic factors in a retrospective study of early-stage hormone-negative breast cancer. Breast Cancer Res. 2015; 17:65. 25935535 10.1186/s13058-015-0575-2 PMC4453198

[44] Ponath V., Kaina B. Death of monocytes through oxidative burst of macrophages and neutrophils: killing in trans. PLoS One. 2017; 12:e0170347. 28099491 10.1371/journal.pone.0170347 PMC5242493

