# Supplementary material for: Sp1 phosphorylation by ATM downregulates BER and promotes cell elimination in response to persistent DNA damage
Source: Nucleic Acids Res. 2017 Dec 27;46(4):1834–46. doi: 10.1093/nar/gkx1291 (PMC5829641; doi:10.1093/nar/gkx1291)
Supplement: Supplementary Data [file gkx1291_supp.pdf]

Sp1 phosphorylation by ATM downregulates BER and promotes cell elimination in response to persistent DNA damage

SALLY C FLETCHER[1], CLAUDIA P GROU[1], ARNAUD J LEGRAND[1], XIN CHEN[1,2], KALLE SODERSTROM[3], MATTIA POLETTO[1,6], GRIGORY L DIANOV[1,4,5,6,7]

[1]CRUK & MRC Oxford Institute for Radiation Oncology, University of Oxford, Department of Oncology, Old Road Campus Research Building, Oxford OX3 7DQ, UK

[2]Department of Marine Technology, College of Ocean, Nantong University, Nantong, China

[3]Nuffield Department of Orthopaedics, Rheumatology and Musculoskeletal Sciences, Botnar Research Centre, University of Oxford, Oxford OX3 7LD, UK.

[4]Institute of Cytology and Genetics, Russian Academy of Sciences, Lavrentyeva 10 Novosibirsk 630090, Russian Federation

[5]Novosibirsk State University, Pirogova 2, Novosibirsk 630090, Russian Federation

[6]Co-corresponding authors

[7]Lead Contact

Supplementary Information

The following file contains supplementary material for the paper "*Sp1 phosphorylation by ATM downregulates BER and promotes cell elimination in response to persistent DNA damage*", by Fletcher SC *et al*. This file is composed of:

- Supplementary methods
- Supplementary figures and relative supplementary figure legends (Two figures)
- Supplementary references

[*]To whom correspondence should be addressed: Tel: +44 1865 617325, Fax: +44 1865 617355, email: grigory.dianov@oncology.ox.ac.uk

SUPPLEMENTARY MATERIALS AND METHODS

siRNA transfections

siRNA transfections were carried out using the Lipofectamine RNAiMAX reagent (Life Technologies) according to the manufacturer's protocol. Unless otherwise indicated, cells were transfected with 30 nM siRNA and analysed 72 hours after transfection. Control transfections were carried out using a non-targeting siRNA (Eurogentec, SR-CL000-005). siRNA oligonucleotides were obtained from Eurogentec. Sequences used were:

Target	Sequence (5' to 3')
ATR	AACCUCCGUGAUGUUGCUUGA
Chk1	GGCUUGGCAACAGUAUUUCGGUAUA
Chk2	AUUGCACUGUCACUAAGCA
DNA-PKcs	CUUUAUGGUGGCCAUGGAG
XRCC1	AGGGAAGAGGAAGUUGGAU
Sp1 #1	AACAGCGUUUCUGCAGCUACC
Sp1 #2	GGUAGCUCUAAGUUUUGAU
XRCC1	AGGGAAGAGGAAGUUGGAU

Real-time PCR (qPCR)

Extraction of total RNA, reverse transcription and qPCR were performed as described elsewhere (1). The comparative CT method was applied for quantification of gene expression; *GAPDH* and *B2M* were used as endogenous controls. Primers used were:

Target	Forward primer (5' to 3')	Reverse primer (5' to 3')
B2M	ATGTCTCGCTCCGTGGCCTTA	ATCTTGGGCTGTGACAAAGTC
BAX	AGCTTCTTGGTGGACGCAT	CAGAGGCGGGGTTTCATC
GAPDH	AGCCACATCGCTCAGACAC	GCCCAATACGACCAAATCC
PUMA (BBC3)	GTAAGGGCAGGAGTCCCAT	GACGACCTCAACGCACAGTA
XRCC1	CTGGGACCGGGTCAAAAT	CAAGCCAAAGGGGGAGTC

XRCC1 ChIP	ATTGGGAGGCGAGGCTA	TCTCCAGAGCGGGAAGAG

Western blot

Whole cell extracts for Western blot were prepared as described previously (2). Secondary antibodies conjugated with Alexa Fluor 680 (Molecular Probes) and IRDye® 800 (Rockland) fluorescent dyes were used for detection. Acquisition and densitometric quantification were carried out using an Odyssey image analysis system (Li-Cor Biosciences). Antibodies used were:

**Target**	**Antibody**
α-tubulin	T6199 – Sigma
ATM	A1106 – Sigma
ATR	ab2905-Abcam
BAX	#5023 – Cell Signaling Technology
Chk1	sc-8408 – Santa Cruz
Chk2	05-649 – Millipore
Cleaved caspase 3 (Asp175)	#9661 – Cell Signaling Technology
Control Rabbit IgG	sc-2027 – Santa Cruz
DNA-PKcs	sc-9051 Santa Cruz
LigIII	Rabbit polyclonal, made in house
pATM (ser1981)	ab81292 – Abcam
pS/pT QG	#6966 – Cell Signaling Technology
pSp1 (ser101)	39757 – Active Motif
Sp1	07-645 – Millipore
XRCC1	MS-1393-P0 – Thermo Scientific
β-actin	ab6276 – Abcam
γH2AX (ser139)	05-636 –Millipore

Supplementary Figures

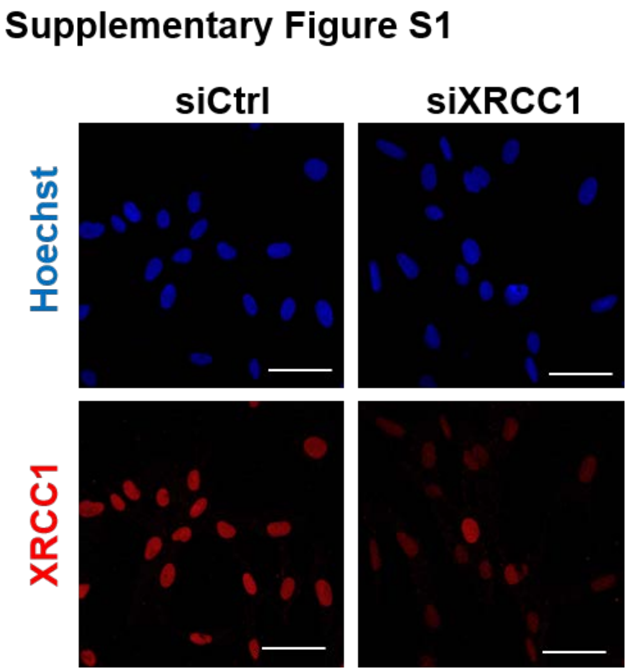

Supplementary Figure S1 – Validation of XRCC1 antibody.

Representative micrographs on cells treated as in with XRCC1 siRNA showing specificity of antibody used for staining. Scale bars 50 µm.

Supplementary Figure 2

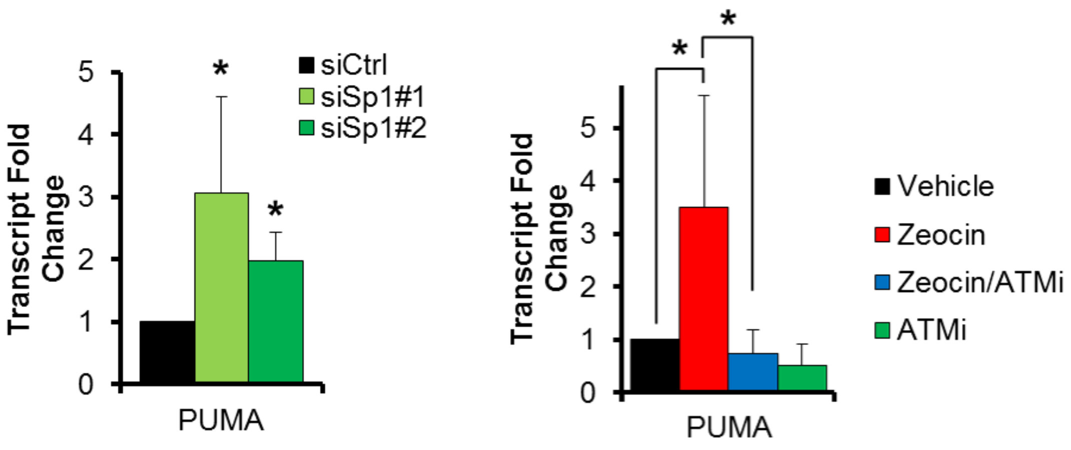

Supplementary Figure S2 – Loss of Sp1 upregulates pro-apoptotic genes.

A qPCR analysis assessing transcription of PUMA (*BBC3*) TIG-1 cells treated with the indicated siRNA (N=3).

B qPCR analysis assessing transcription of PUMA (*BBC3*) in fibroblasts treated with zeocin (50 µg/ml, 6 h). An ATM inhibitor (ATMi - KU60019, 10 µM) was added where indicated. Induction of *Bax* is completely prevented by ATM inhibition (N=4).

Data information: data are reported as mean ± SD from the indicated number (N) of independent experiments *:$p<0.05$.

Supplementary References:

1. Poletto, M., Legrand, A.J., Fletcher, S.C. and Dianov, G.L. (2016) p53 coordinates base excision repair to prevent genomic instability. *Nucleic Acids Res*, **44**, 3165-3175.
2. Orlando, G., Khoronenkova, S.V., Dianova, II, Parsons, J.L. and Dianov, G.L. (2014) ARF induction in response to DNA strand breaks is regulated by PARP1. *Nucleic Acids Res*, **42**, 2320-2329.
